# Influence of Instant Controlled Pressure Drop (DIC) on Allergenic Potential of Tree Nuts

**DOI:** 10.3390/molecules25071742

**Published:** 2020-04-10

**Authors:** Fatima Vicente, Africa Sanchiz, Rosa Rodríguez-Pérez, Maria Pedrosa, Santiago Quirce, Joseph Haddad, Colette Besombes, Rosario Linacero, Karim Allaf, Carmen Cuadrado

**Affiliations:** 1 Food Technology DepartmentSGIT-INIA, Ctra. La Coruña Km. 7.5, 28040 Madrid, Spain; fatima.vicente.martin@gmail.com (F.V.); africa.sanchiz@inia.es (A.S.); 2 Allergy Service, University Hospital La Paz, IdiPAZ, 28046 Madrid, Spain; rosa.rodriguez@idipaz.es (R.R.-P.); m.pedrosa.d@gmail.com (M.P.); squirce@gmail.com (S.Q.); 3 Laboratory Engineering Science for Environment (UMR 7356 CNRS), La Rochelle University, venue Michel Crepeau, 17042 La Rochelle, France; jhhaddad75@gmail.com (J.H.); colette.besombes@univ-lr.fr (C.B.); kallaf@univ-lr.fr (K.A.); 4 Genetics, Physiology and Microbiology Department, Biology Faculty, Complutense University, 28040 Madrid, Spain, 28040 Madrid, Spain; charolin@ucm.es

**Keywords:** pistachio, cashew, allergens, instant controlled depressurization (DIC) processing, pressure processing, thermal processing

## Abstract

Pistachio and cashew contain allergenic proteins, which causes them to be removed from the diet of allergic people. Previous studies have demonstrated that food processing (thermal and non-thermal) can produce structural and/or conformational changes in proteins by altering their allergenic capacity. In this study, the influence of instant controlled pressure drop (DIC) on pistachio and cashew allergenic capacity has been studied. Western blot was carried out using IgG anti-11S and anti-2S and IgE antibodies from sera of patients sensitized to pistachio and cashew. DIC processing causes changes in the electrophoretic pattern, reducing the number and intensity of protein bands, as the pressure and temperature treatment increment, which results in a remarkable decrease in detection of potentially allergenic proteins. The harshest conditions of DIC (7 bar, 120 s) markedly reduce the immunodetection of allergenic proteins, not only by using IgG (anti 11S and anti 2S) but also when IgE sera from sensitized patients were used for Western blots. Such immunodetection is more affected in pistachio than in cashew nuts, but is not completely removed. Therefore, cashew proteins are possibly more resistant than pistachio proteins. According these findings, instant controlled pressure drop (DIC) can be considered a suitable technique in order to obtain hypoallergenic tree nut flour to be used in the food industry.

## 1. Introduction

According to the EAACI (European Academy of Allergy and Clinical Immunology), food allergy is a public health problem that affects approximately 4%–7% of young children in Europe [1]. More than 120 foods have been described as causing food allergies; however, according to European Regulation 1169/2011, only 14 allergens have been classified as requiring mandatory declaration on labeling: cereals containing gluten, crustaceans, eggs, fish, peanuts, soybeans, dairy products, nuts, celery, mustard, sesame grains, mollusks, lupines and sulphites.

Pistachio and cashew nuts have a high number of beneficial nutritional properties for human health. Therefore, their consumption contributes with healthy benefits [2]. Several trials have shown that pistachios promote blood lipid profiles favorable to the heart and can prevent cardiovascular disease. In addition, they help to maintain healthy antioxidant and anti-inflammatory activity, glycemic control and endothelial function [3]. Cashew and its derivatives have been traditionally used for their medical properties, such as antimicrobial, anticancer and anti-inflammatory capacity [3], reduction of hypertension and treatment of gastrointestinal disorders [4]. At the nutritional level both nuts have a high content of fatty acids, mainly oleic acid (18: 1) and linoleic acid (18: 2) (approximately 45%–50% of the seed). Around 20% of the seed of these nuts is protein and highlights the presence of all essential amino acids. It also highlights the presence of carbohydrates and fiber, the latter being very notable in the case of pistachio relative to other nuts. In addition, they are a source of vitamins, minerals and pigments [2].

Pistachio allergy is rare in the US. However, according Costa el al.’s data [5], it seems that the prevalence of pistachio allergy is increasing due to increased production and consumption, the European Union being the second largest consumer of pistachio after Turkey. So far, five proteins have been identified and characterized as food allergens in pistachio Pis v 1 (2S albumin), Pis v 2 (11S legumin), Pis v 3 (7S vicilin) and Pis v 5 (11S legumin), are seed storage proteins that belong to the prolamine and cupin superfamilies [6,7,8], while Pis v 4 is classified as a defense protein of the iron/manganese superoxide dismutase protein family [9]. Pis v1 and Pis v2 are considered the major allergens [5].

Regarding cashew nut reactions and symptoms, several US studies have reported that it is the second most common cause of nut allergy, together with the walnut, presenting a frequency of 20% [10]. Cashew allergy seems to be increasing epidemiologically in recent years, especially in northern Europe, where allergy ranges from 5% to 20% depending on the country [11]. So far, three allergenic proteins in cashew have been identified and characterized: two of them belong to the cupin superfamily (Ana or 1 and Ana or 2) and one to the prolamine superfamily (Ana or 3) [12,13,14]. It is estimated that more than 80% of patients with pistachio allergy have cross-reactivity to other nuts, such as peanuts, walnuts, chestnuts, almonds, pine nuts and specially cashew, given the identity between Pis v 1 and Pis v 2 sequences with Ana o 3 and Ana o 2 (64% and 48% respectively) [6]. 

Many authors have documented the possibility of using technological food processing to modify the physical–chemical properties of proteins; their structure, function, digestibility and solubility could be altered, which could in turn alter their immunoreactivity. Any process that modifies the structure of a protein could be able to interfere with the ability of antibody binding. The degree of alteration of the allergenicity depends on many parameters: method used, processing conditions (time, intensity or environment), and type of food and allergenic capacity [15]. There are different types of processing: non-thermal treatments, such as enzymatic processing, achieving a reduction in immunoreactivity against IgE antibodies [16], and thermal treatments, such as cooking, baking, frying and roasting among others. Cooking has little effect on nut allergens, although recent studies have concluded that some peanut allergens are transferred from the seed to the cooking water during this type of processing [15]. When heat and pressure are combined in particular conditions, such as autoclave at 138 °C/2.56 atm for 15 or 30 min, immunoreactivity of IgE antibodies in peanut, pistachio, cashew, lentil or chickpea is importantly reduced [16,17,18]. In controlled instant depressurization (DIC) treatment, high temperatures, up to 180 °C, and pressure, up to 8 bars, are applied for short periods of time (from a few seconds to a few minutes. Applying this treatment, similar reductions in IgE immunoreactivity have been observed to those obtained in the harshest autoclave treatments in different raw and roasted legumes [19,20]. An advantage of DIC over other processing technologies is that pressures higher than other treatments are reached (up to 8 bars, while autoclave is reached at most at 3 bars) and that the exposure to the treatment develops for very short periods of time, preventing damage caused by heat that can affect other properties, apart from allergenic capacity [21,22]. However, there are no fixed rules concerning the effect of processing on the allergenic capacity of food, since some epitopes can be eliminated, others can remain unchanged or even new ones can be generated (neo-allergens) of greater potency [16,17,23]. The understanding of the potential effects of food processing on their allergenic properties constitutes an active area of investigation.

The main objective of this work is to evaluate the influence of instant controlled pressure drop (DIC) on the protein profile and allergenic potential of pistachio and cashew nuts. 

## 2. Results and Discussion

Food processing can modify allergenic capacity by altering the physicochemical properties of its proteins [24]. Treatments that combine heat and pressure have proven effective in reducing the allergenic capacity of many animal and plant-based foods. In addition, it has been shown that heat treatment can alter other properties of food, which may affect its techno-functional properties [25]. That is why the application of DIC is proposed, which combines pressure and temperature at short times (1–2 min), as a treatment with great potential to reduce the allergenic capacity of these two nuts without affecting other properties.

### 2.1. Optimization of DIC Treatments

**Figure 1:**
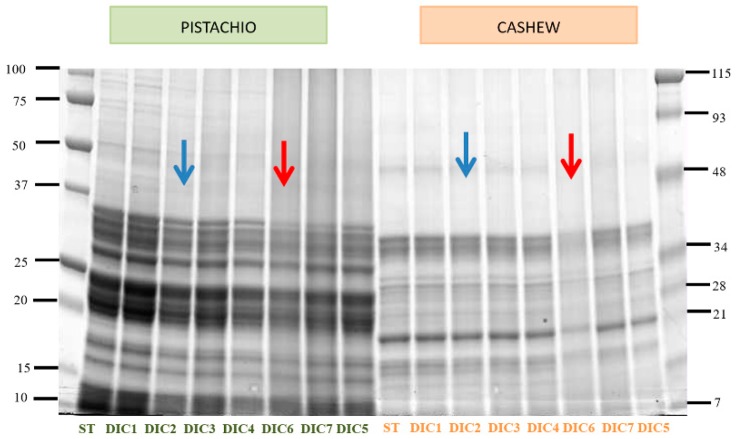
SDS-PAGE (12%) of pistachio flour (marked in green) and cashew nut (marked in orange). (20µg of protein per lane). The red and blue arrows indicate the selected treatments by presenting variations in the band pattern. Precision Plus (right, P+) and Broad Range (left) molecular weight markers were used (Bio-Rad).

For the selection of the most effective DIC treatment conditions, an Sodium dodecyl sulfate polyacrylamide gel electrophoresis (SDS-PAGE) electrophoresis of 13 pistachio and cashew flours processed with different pressures and time conditions was carried out, from 3 bar, 30 s to 7 bar, 75 s (data not shown). From that electrophoresis, seven samples showing changes in the protein profile were selected (DIC 1 to DIC 7) whose operation conditions are detailed in Table 3 in Section 3.3 of Materials and Methods The SDS-PAGE electrophoretic profile of the control (ST) and these seven treated pistachio and cashew nuts (DIC1-DIC7) samples is shown in Figure 1. 

**Figure 2:**
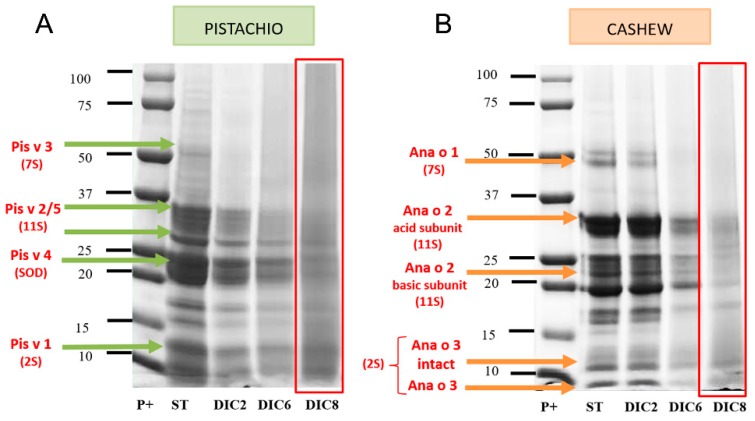
SDS-PAGE (4%–20%) of control (ST) and DIC (instant controlled depressurization)-treated pistachio flour (**A**) and cashew (**B**) (20 µg of protein/lane). The green and orange arrows indicate the allergenic bands described in pistachio and cashew respectively.

The protein pattern hardly varies after applying the different DIC treatments. The band intensity is reduced in the case of the DIC6 treatment (6.4 bar, 107 s, red arrow), and in the DIC2 treatment (3.6 bar, 107 s, blue arrow) which reduces the band intensity only in the case of pistachio. Previous studies have also pointed out that thermal and pressure processing affects pistachio proteins more than cashew nuts [16,18]. In order to obtain a more effective DIC treatment in the reduction of protein intensity, a third treatment was developed with conditions of greater pressure and time (DIC8 7 bar, 120 s). When analyzing DIC2, DIC6, DIC7 and DIC8, a greater effect was observed when applying 7 bars for 120 s (DIC8) compared to 7 bars for 75 s (DIC7) (Figure 2). Similar results were observed by Cuadrado et al. [19] in peanuts and soybean, in which, at the same pressure, lower band intensity was observed with longer exposure times. Therefore, it can be deduced that both pressure and exposure time are determinant for protein modification.

### 2.2. Analytical Composition

In order to know the effect that the different DIC treatments have on the protein content, different tests were carried out to assess the total and soluble protein content in each of them. The determination of the total protein was analyzed by RC-DC ((Reducing Agent and Detergent Compatible)^®^ Protein Assay (Bio-Rad) and by the Dumas method (LECO^®^ analysis) using 5.3 as the nitrogen-to-protein conversion factor [26], whereas the soluble protein content was determined by the Bradford method (Table 1).

The total protein reduction in DIC8 was greater in pistachio (44%) than in cashew (31%). Regarding soluble protein, DIC8 treatment caused a reduction of 82% in pistachio and 72% in cashew. According to Cabanillas et al. [27], the modifications produced by heat and pressure in the soluble protein fraction can be considered representative of the changes produced in the same treatments in the total protein. The value of soluble protein is also lower than that of total protein, because in most cases the solubility decreases after processing due to the formation of protein aggregates of reduced solubility [28]. Cuadrado et al. [16] also observed similar values of soluble protein (around 33 and 42 g/100 g) in pistachio and cashew flours treated with cooking and with autoclave, in addition to little variation in the soluble protein content.

### 2.3. Electrophoretic Analysis

Figure 2 shows the SDS-PAGE electrophoretic profile of treated pistachio and cashew flours selected to carry out the immunoassays (DIC 2, DIC6 and DIC8) in addition to the control samples (ST).

The electrophoretic profile of the flours shows a band pattern that ranges between 10 and 100 kDa in the case of pistachio, the most intense bands being located around 10–15 kDa and 20–37 kDa. In the case of cashew nuts, the band pattern ranges from 10 to 80 kDa, finding the most intense bands around 20 kDa and 37 kDa.

The positions compatible with the allergens so far described are shown with arrows; in the case of untreated pistachio (Pis v 1, Pis v 2, Pis v 3, Pis v 4 and Pis v 5), the bands located around 10–15 kDa stand out, which could correspond to Pis v 1 (2S); the bands around 25 and 37 kDa are compatible with the positions of the allergenic proteins Pis v 2 and Pis v 5, both 11S. Regarding Pis v 3 (7S), it can be located around 50 kDa, although the band does not appreciate intensely and, finally, the bands observed between 20–25 kDa would correspond to the area where the allergen Pis v 4 (SOD) migrates. 

Regarding the effect of the DIC treatment, having as reference the control sample, the number and intensity of bands decrease as the treatment conditions become more drastic (higher pressure and time). The most effective treatment is DIC8, in which both the number of bands and the intensity decrease. The most resistant bands are those located around 10–15 kDa, possibly a 2S albumin and those located around 20, 32 and 36 kDa, corresponding to SOD and 11S legumes, respectively.

In the case of the untreated cashew nut (Ana o 1, Ana o 2 and Ana o 3) in the 50 kDa zone, a band is observed that could correspond to Ana o 1. The bands located around 33 and 22 kDa are compatible with the positions of the subunits (acidic and basic) of Ana o 2. Finally, the band observed around 10 kDa would coincide with the position of the intact subunit of the allergen Ana o 3 and the band around 7 kDa with that of the major subunit of this allergen. As in the case of pistachio, DIC treatment reduces band intensity as pressure and time increase, and the most effective treatment is DIC8 in which both the number and intensity of bands is reduced, although there are still 10–15 kDa proteins compatible with 2S albumin, around 16 kDa (protein not coinciding with any of the described as allergens in cashew nuts) and the possible acid and basic subunits of the 11S legumin located at 20 and 30 kDa.

Guillamón et al. [29] and Cuadrado et al. [19], also studied the effect of DIC at 3 bar and 6 bar for 1 and 3 min in flours of different plant species: lupine, chickpea, lentil, soybeans and raw and roasted peanuts. After the most drastic treatment, none of the lupine allergens was detected. In the case of peanut, the intensity of the electrophoretic pattern decreases without disappearing in the range 15–65 kDa. In lentil and chickpea, the band intensity decreased relative to the control, although changes were hardly observed between the different treatments; this fact leads to the deduction that the conditions of the DIC8 treatment are more favorable for the decrease in the number and intensity of protein bands. However, even under these conditions, protein bands are still observed, especially those with low molecular weight. 

### 2.4. Immunodetection

#### 2.4.1. Immunodetection with Anti-2S and Anti-11S IgG

**Figure 3:**
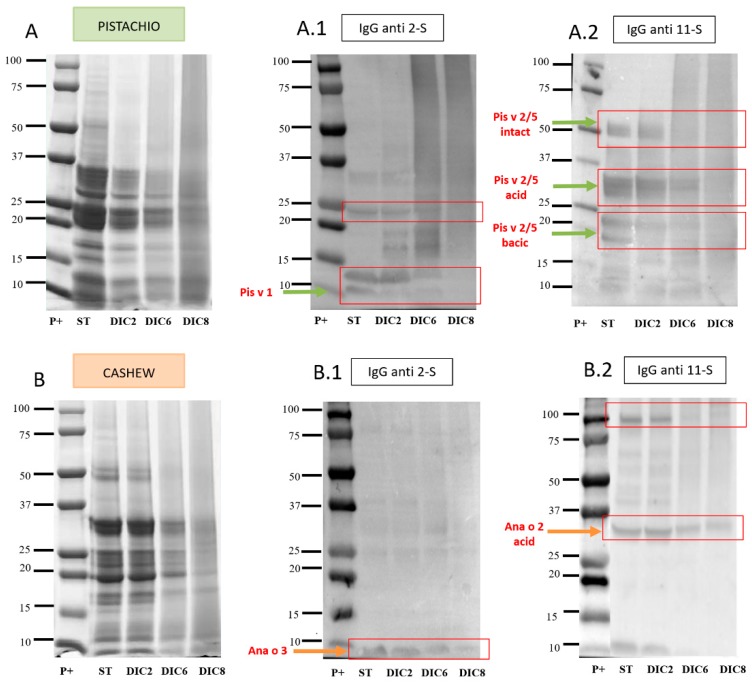
SDS-PAGE (4%–20%) and IgG immunoblots of pistachio (**A**) and cashew (**B**). Immunoblots with IgG anti 2-S (**A.1** y **B.1**) and anti 11-S (**A.2** y **B.2**). Proteins recognized are marked in red and indicated by arrows. (20 µg protein/lane).

To deepen the study of the effect of the DIC treatment, at different pressures and times, immunodetection by Western blot (WB) of potential allergenic proteins was carried out in the defatted flours of the two nuts, using anti-2S and anti-11S IgG antibodies. The results of these analyzes are shown in Figure 3.

Figure 3A.1 shows immunodetection with the anti-2S IgG antibody in control pistachio flours (ST) and treated with DIC. The binding of the antibody to bands located in the 10 kDa zone could correspond to Pis v 1 (2S albumin). This protein band is still present in the DIC2 and DIC6 treatment, but in DIC8 becomes almost undetectable. The 11S globulins are formed by two subunits: acidic (30 kDa) and basic (20 kDa) [10,30]. The results of incubation with anti-11S IgG antibody are observed in Figure 3A.2. In the control sample, three bands located around 50 kDa, 30 kDa and 20 kDa stand out, compatible with pistachio legumins (11S) proteins: intact Pis v2/5 and the acid and basic subunits, respectively. In the case of DIC6 and DIC8, the bands that could correspond to the acid and basic subunits are almost imperceptible, and the intact Pis v2/5 disappears. 

Immunodetection with anti-2S in cashew flour is shown in Figure 3B.1. In the case of the control and the three DIC treatments, a band around 10 kDa was observed compatible with Ana or 3 albumin; it is remarkable that it is still present even in the most drastic treatment (DIC8). Immunodetection with anti-11S antibody in cashew flours (Figure 3B.2) highlighted the presence of bands around 30 kDa, which could correspond to the acid subunit of the legume Ana or 2; this band is decreasing in intensity.

From these immunodetection profiles can be deduced that cashew allergens are more resistant to the treatment applied than those of pistachio, which are almost completely eliminated in DIC8, while in cashew nuts, although they decrease in intensity, they are still present. 

#### 2.4.2. Immunodetection with IgE from Human Sera

**Figure 4:**
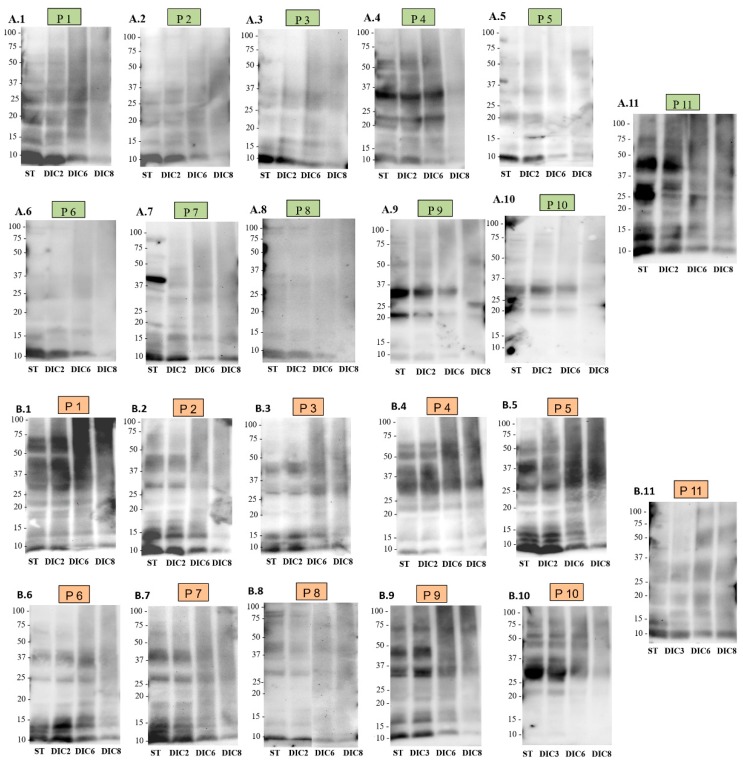
IgE immunoblot of proteins of pistachio (**A**) and cashew (**B**) of untreated control (ST) and DIC-treated samples (20 µg protein/lane). IgE immunoblots were carried out using individual sera from 11 patients allergic to pistachio (**A.1 to A.11**) and cashew (**B.1 to B.11**) (**P1**–**P11**).

In order to further investigate the effect of DIC treatment on the allergenic capacity of pistachio and cashew, Western blots were carried out using individual sera from 11 Spanish patients sensitized to both nuts. Previous studies on immunodetection in pistachio and cashew flour were carried out using a mixture of sera (pool), instead of individual sera, which showed less variability in the band pattern [18]. The sera of the patients used in the present study belong to children with sensitivity to both pistachio and cashew nuts, some with a well-characterized clinical history that present from acute reactions (edema or hives) to serious adverse reactions (anaphylaxis), others present sensitization to both nuts, but without having consumed them (Table 2). The results of such immunodetection are shown in Figure 4 and Figure S1 of supplementary material.

In Figure 4A.1–A.11, the WB with human sera in pistachio control and processed flours are shown. Although individual differences are observed in the pattern recognized by the sera, all patients recognize 2S albumin (Pis v 1), and all except P6 and P8, recognize 11S legumin. Regarding the effect of DIC treatment, it is noteworthy that DIC8 significantly reduces the immunodetection of 2S with IgE from human sera, but without removing it completely, since a slight band around 10 kDa is still observed in all patients except P8 and P6. Regarding 11S legumins, DIC8 treatment is able to reduce them, even disappearing as in P1–P4 and P7.

In Figure 4B.1–B.11 is the WB with human sera in cashew control and treated flours. Patient 1 (Figure 4B.1) recognizes bands around 10, 20, 30 and 50 kDa, compatible with the immunodetection of 2S and 11S proteins, Ana o 3, Ana o 2 acidic, basic and intact, although the 50 kDa could also correspond to Ana o 1. A similar pattern is observed in patients 4, 5 and 10. Patient 2 recognizes bands of approximately 10 kDa (possibly Ana o 3) and 30 kDa (acid subunit of Ana o 2). Patients P3, P6, P7 and P11 have an immunoreactivity similar to that of P2. Regarding P8 and P9, they have a different band pattern, just as the rest of the sera recognize a band of about 10 kDa compatible with 2S, a pair of bands around 25–37 kDa, which could correspond to the acid and basic subunits of Ana o 2 legumin and a 75 kDa protein, which does not correspond to any of the allergens described in cashew nuts and is therefore unknown.

As in pistachio, there is variation in the immunoreactive profile of patients against cashew. All patients showed detection of 2S albumin and 11S legumin, being able to recognize the two subunits of Ana o 2, and 4 out of the 11 patients (P1, P4, P5 and P10) might be recognizing Ana o 2 intact, located around 50 kDa, although this protein is also compatible with Ana o 1. Regarding the effect of treatments, the presence of protein degradation is remarkable in the area of high molecular weight of patients 1, 5 and 9, in the last two lanes. As the treatment becomes more intense, the band intensity tends to decrease, but without eliminating the possible Ana o 3 albumin, even in the DIC8 treatment. Regarding the bands compatible with legumins, they tend to disappear in DIC8, although not in all cases; in patients 3, 4 and 11, the possible acid and basic subunits of Ana o 2 are still detected.

Patterns similar to those obtained in this study have been observed by Blanco et al. [31] also using sera from Spanish patients; in the case of pistachio, 13–33 kDa proteins were detected, and in the case of cashew nut around 19–50 kDa were. In previous studies, in which reactivity against IgE in pistachio and cashew flours was evaluated using individual sera of North American origin, individual differences were also observed in each of the immunological patterns [16]. In that case, the immunodetection of bands focused on areas of molecular weight ranging from 20 to 100 kDa, while, in the results of the present study, the detected proteins do not exceed 60 kDa. This indicates differences in immunoreactivity patterns not only among individuals of the same population, but also among populations based on their geographical origin (Spanish or North American) [32].

It should also be noted that, although there are differences between individuals, they all recognize 2S albumins, both pistachio (Pis v 1) and cashew (Ana o 3), except P10. Previous studies have already described the high reactivity of the albumin of these two nuts; Pis v 1 is a major allergen that has been shown to be detected by more than 50% of sera from pistachio-allergic patients [5]. Regarding Ana o 3, it has been determined that it is detected by 93% of children allergic to cashew nuts, becoming a clinical indicator of cashew allergy, being even more specific than the whole extract [33]. Regarding the effect of DIC on this type of protein, its high resistance stands out, as already mentioned above, and was observed in the anti-2S IgG immunodetection (Figure 3(A.1,B.1)) since, although the recognition of these proteins decreases in all patients as the time and pressure of the treatment increases, the band does not disappear. However, in the case of immunodetection of cashew nut proteins with human sera, although the band intensity decreases, there is no such notable reduction compared to control. In addition, it should be noted that almost all patients recognize the 2S albumin of both nuts, probably due to the close botanical relationship between them. Therefore, it is expected that individuals who react to the first also do so to the second, as observed. Recent studies by Bueno-Díaz et al. [34] prove the existence of a very high sensitivity to both allergens, without the appearance of cross-reactivity with other 2S albumins.

Regarding the 11S legumes, they are not detected in all patients: in the case of pistachio in 8 out of 11 patients and in all patients in the case of cashew. In addition, the detected band intensity is quite low compared to those of 2S albumins. Regarding the effect of DIC on this type of protein, it is noted that they are less resistant than 2S albumins, as has also been observed in the anti-11S IgG blots (Figure 3(A.2,B.2)), since, after DIC8 treatment, detection of the corresponding bands was not possible for the majority of patients. Previous studies have shown that 11S legumes are denaturalized at 94 °C, leading to the conclusion that DIC treatment interferes with antibody binding capacity [16]. In addition, the effect of DIC8 treatment is more effective in pistachio legumins (Pis v 2/5) than in cashew legumins. From our point of view, aggregation does not explain the extensive modifications of allergenic proteins observed after processing, especially of DIC. The present data are more in agreement with extensive degradation of the proteins, which would produce short peptides situated in the low MW portion of the gels (causing the smear observed in the processed lanes in Figure 2, Figure 3 and Figure 4) or even so fragmented that they could not be retained by the gel. This could explain the disappearance of many IgE-immunoreactive band proteins and is supported by a large number of published studies confirming it [15]. 

Currently, once a food allergy has been diagnosed, the only treatment is the elimination from the diet of the causative foods, which leads to nutritional and therefore health problems [34]. Several studies have proposed the use of food peptides with reduced immunoreactivity as a strategy to develop immunotherapy against allergies [35], so it is interesting to apply processing techniques other than those already studied. Although there is little information on the effect of DIC in food allergens of nuts, other authors have described the high efficacy of this treatment in the elimination of in-vitro immunoreactivity of other plant species, such as lupine [29], in which DIC treatment at 6 bar, 3 min is able to eliminate IgE immunodetection almost completely. In the case of peanuts, DIC treatment at the same pressure and time conditions generates a remarkable reduction in 65 kDa protein bands and eliminates the immunoreaction of bands less than 20 kDa. In soybeans, all immunoreactive proteins were eliminated, and in chickpea, immunoreaction with human sera detected a lower number of bands than the control treatment, and the intensity of them also decreased. Regarding the effect of DIC treatment on pistachio and cashew nut allergens, it seems to be effective, although it is not capable of abolishing its allergenic capacity. DIC treatment at 7b for 120 s resulted in a reduction of 75% of immunoreactive bands in pistachio and 67.2% in cashew compared to the untreated samples (Figure S2 of supplementary material). Previous studies [18] have shown that heat treatment by cooking for 30 and 60 min does not reduce the detection of allergenic proteins by human sera, using a pool of sera from Spanish patients. However, in the same study it is observed how the treatment of pressure and temperature reduces the detection of allergenic proteins, even eliminating the detection completely under the strongest conditions of time and temperature (138 °C, 30 min). Similar results were observed by Cuadrado et al. [16] when applying the same treatment together with an enzymatic hydrolysis with proteases, using seven individual sera from North American patients. Date suggest, therefore, that the conditions of DIC treatment applied are less effective for the reduction of immunoreactivity in allergic patients than autoclave treatments. However, the fact that the in vitro detection of allergens decreases because of food processing does not always imply a reduction in the in vivo allergenicity. To complete the analysis, it would be necessary to perform tests using in vitro allergy models such as the human basophil activation test (BAT) or humanized basophil cell lines (RBL), animal models or clinical trials, using skin tests (SPT). The results of this study could be relevant, since the use of peptides with hypoallergenic properties has been proposed as a strategy to develop immunotherapy against food allergies. After a mass spectrometry characterization, these peptides might have the ability to activate the immune system but without triggering the allergic response [36].

### 2.5. Proteins identification by LC/MS/MS

Cashew and pistachio proteins from untreated and controlled instant depressurization were loaded in a 4%–20% Tris-glycine gel in order to identify the band proteins. The major bands were tryptic digested in order to carry out the analysis by mass spectroscopy (LC/MS/MS). Finally, eight bands of pistachio and nine in cashew were manually excised for MALDI-TOF/TOF identification (Figure S3 of supplementary material). The table summarizes the polypeptide identification data of these pistachio and cashew samples. A peptide mass fingerprint search allowed us to identify all the bands of pistachio (five from untreated and three in the DIC8 for 7-bar, 120-s samples) as Pis v 1, Pis v 3, Pis v 2 or Pis v 5. The peptides identified from bands 1 and 2 from untreated pistachio samples were Pis v 2, Pis v 5 and Pis v 3. Bands 3, 4, 3′ and 4′ were identified as Pis v 2. Pis v 1 was detected in bands 5 and 5′. Band 6 (50 kDa) had peptides from Ana o 1 and Ana o 2. Bands 7, 8, 7′, 8′ and 9′ were identified as Ana o 2. Ana o 3 was detected in bands 9, 10 and 10′ but not in band 9′, suggesting that it is degraded into smaller fragments. These findings confirm the compatible location of pistachio and cashew allergens proposed in Figure 2 and agree with the protein identification by LC/MS/MS previously reported in the literature [5,11]. The results of protein identification by MS analysis (Figure S3 of supplementary material) indicate that some allergens are being degraded, explaining the reduction of immunoreactive proteins over DIC treatment in agreement with Cuadrado et al., [16]. Even at the harshest conditions (7 bars, 120 s) this DIC processing produced resistant peptides, indicating that some fragments of pistachio and cashew allergens survive this pressured thermal treatment.

## 3. Materials and Methods

### 3.1. Plant Material

Pistachio (*Pistachia vera* L. var. Kerman) and cashew nuts (*Anacardium occidentale* L. type 320) were obtained from the germplasm bank of IRTA Mas Bover (Institute of Agrifood Research Technology, Tarragona, Spain) and Productos Manzanares SL (Cuenca, Spain) respectively. 

### 3.2. Human Sera

Anonym individual sera were chosen from 11 patients from the Hospital La Paz in Madrid sensitized to both nuts, positive IgE-CAP (>0.35 kU/L) whose IgE values ranged from 15.4 to > 100 kU/L for pistachio and 10.1 to > 100 kU/L for cashew nuts as quantified by using the CAP-FEIA (fluorescent enzyme immunoassay) system (Pharmacia Diagnostic) (Table 2). A serum from a patient with specific IgE to Anisakis ssp. (9.09 kU/L), specific IgE < 0.35 kU/L to pistachio and cashew and a total serum IgE value of 53.4 kU/L was used as a negative control (Figure S1 in the supplementary material).

### 3.3. Controlled Instant Depressurization Treatments (DIC)

Both nuts were subjected to instant controlled depressurization (DIC) treatments carried out at the University of La Rochelle (Laboratoire Maîtrise des Technologies Agro-Industrielles, La Rochelle, France). DIC treatment was carried out in duplicate following a factorial experimental design previously described [21,22]. Briefly, the moistened whole nuts are placed in a processing chamber and exposed to steam pressure (up to 8 bar) at high temperature (up to 170 °C), over a relatively short time (few seconds to some minutes). This high-temperature–short time stage is followed by an instant pressure drop towards a vacuum at about 50 mbar. This abrupt pressure drops, at a rate ∆P/∆t higher than 5 bar s^−1^, simultaneously provokes an auto-vaporization of a part of the water in the product, and an instantaneous cooling of the products, which stops thermal degradation. After the DIC treatment of pistachio and cashew nuts, a total of 13 samples were obtained that include treatments at different pressure and time conditions: from a minimum point of 3 bar, 30 s; up to a maximum of 7 bar, 75 s; being the central point of 5 bar, 75 s. The electrophoretic profile of these 13 samples was analyzed and the 7 that presented changes in the protein pattern were selected (DIC1-DIC7, Table 3). Two treatments (DIC2 and DIC6) out of seven were selected to compare the possible reduction in the number and intensity of protein bands. Posteriori, another DIC treatment was carried out with more pressure and time (DIC8, 7 bar and 120 s), in order to check if the effectiveness increased and a greater protein modification was obtained, which was also selected. Therefore, three samples, DIC2, DIC6 and DIC8, were selected to carry out the electrophoretic and immunodetection analysis.

All processed and control samples were grounded using a Thermomix cooking robot (Thermomix TM-31, Vorweck, Wuppertal, Germany) for approximately 10 s, thus obtaining cashew and pistachio flours, both treated and untreated. The flours were defatted with *n*-hexane (34 mL/g dm). The defatted flour was passed through a 1 mm mesh. This flour was used in subsequent experiments and assessments.

### 3.4. Protein Separation and Immunoblot

Defatted and milled flours of untreated (control) and DIC-treated tree nuts were used for these analyses. SDS-PAGE was performed according to the Laemmli protocol and the samples were extracted with SDS Laemmli sample buffer [37] using 12% polyacrylamide gels (Criterium, Bio-Rad, Hercules, CA, USA) and 4%–20% gradient polyacrylamide gels (Miniprotean, Bio-Rad, Hercules, CA, USA)) as previously reported [16]. Staining was performed with Coomassie Brilliant Blue R-250. The gels were analyzed with the Quantity One (Bio-Rad, Hercules, CA, USA) program. Total protein content was determined by RC-DC (Reducing Agent and Detergent Compatible, Bio-Rad, Hercules, CA, USA) method, based on the method of Lowry et al. [38], and the nitrogen contents of the samples were determined by LECO analysis according to standard procedures based on the Dumas method [26]. The total protein content was calculated as N “x” 5.3 [26]. The analyses were carried out in duplicate and the results summarized in Table 3. The protein content of processed samples was higher than in the raw ones. This could be related to the reduction of dry matter in these processed samples. 

#### 3.4.1. Immunodetection with IgG Antibodies

For western blot, proteins were transferred to polyvinylidene difluoride (PVDF) membranes (Millipore Corp., Bedford, MA, USA) using a semi-dry transfer system (iBlot 2Dry Blotting System, Invitrogen, Carlsbad, CA, USA). Blocking was carried out for 1 h, at room temperature in PBS plus 0.5% Tween-20 (PBST) containing 3% milk (blocking solution). IgG mouse anti-11S (dilution 1:10000) and anti-2S (1:25000) were diluted in blocking solution and incubated with PVDF membranes for 1 h. Membranes were washed and then treated with alkaline phosphatase (AP) conjugated goat anti-mouse antibody (1:5000) (Sigma, Saint Louis, MO, USA) diluted in blocking solution. Detection was achieved by means of BCIP/NBT substrate (Sigma, Saint Louis, MO, USA). The signal was measured using ChemiDoc (Bio-Rad, Hercules, CA, USA). For IgE western blot, proteins were transferred to a polyvinylidene difluoride (PVDF) membrane. After that, membranes were incubated overnight at 4 °C with pool sera from 11 patients with tree nut allergies, washed and then treated with horseradish peroxidase (HRP) conjugated mouse anti-human IgE (1:10000 dilution for 30 min at RT) (Sigma, Saint Louis, MO, USA). Detection of IgE-binding proteins was achieved by means of enhanced chemiluminescence, according to the manufacturer’s instructions (Thermo Scientific, Waltham, MA, USA). The signal was measured using the CCD camera system of ChemiDoc (Bio-Rad, Hercules, CA, USA). 

#### 3.4.2. Immunodetection with IgE of Human Sera

After electrophoresis, the gels were transferred to PVDF membrane (Millipore Corp., Bedford, MA, USA) at 20 V, 7 min, using a semi-dry transfer system (iBlot 2Dry Blotting System, Invitrogen, Carlsbad, CA, USA). After the transfer, the membrane was blocked with 2% blocking solution of milk in PBST for 30 min with stirring. To remove excess blocking solution, the membranes were washed three times for 5 min in PBST. Then, the membranes were incubated with the 11 individual sera (dilution 1:10 or 1:20 in PBST), for 16 h, at 4 °C. After washing three times with PBST, they were incubated with the secondary antibody (Mouse Anti-Human IgE Fc-HRP, Southern Biotech, Birmingham, AL, USA) (diluted 1:10,000 in 2% blocking solution and DynaLight (1:62500) (Precision protein Strep Tractin-HRP Conjugate, Invitrogen, Carlsbad, CA, USA). The membranes were washed three times with PBST and a final wash with PBS was given. In this case, chemiluminescence was used for membrane development. The substrate Pierce ECL 2 (Thermo Scientific, Waltham, MA, USA) was used under the conditions indicated by the manufacturer. The membrane was also scanned in ChemiDoc (Bio-Rad, Hercules, CA, USA) and the images were analyzed with ImageLab software (Bio-Rad, Hercules, CA, USA) at different exposure times.

### 3.5. Protein Identification by MS and Data Base Search

Selected immunoreactive pistachio and cashew proteins, untreated and treated by controlled instant depressurization (DIC 8 at 7 bars, 120 s), were analyzed by MS and database search in order to determine their identification. The gel spots of interest were manually excised from gels. Proteins selected for analysis were reduced in gel, alkylated and digested with trypsin following Sechi and Chait [39]. Briefly, the samples were reduced with 10 mM dithioerytritol in 25 mM ammonium bicarbonate for 30 min at 56 °C and subsequently alkylated with 25 mM iodoacetamide in 25 mM ammonium bicarbonate for 15 min in the dark. Finally, samples were digested with 12.5 ng/µL sequencing grade trypsin (Roche Molecular Biochemicals, Indianapolis, IN, USA) in 25 mM ammonium bicarbonate (pH 8,5) overnight at 37 °C. After digestion, the supernatant was collected and 1 µL was spotted onto a MALDI target plate and allowed to air dry at room temperature. Then, 0.7 µL of a 3 mg/mL of α-cyano-4-hydroxy-cinnamic acid matrix (Sigma, Saint Louis, MO, USA) in 50% acetonitrile was added to the dried peptide digest spots and allowed to air dry again at room temperature. MALDI-TOF MS analyses were performed in a 4800 Plus Proteomics Analyzer MALDI-TOF/TOF mass spectrometer (Applied Biosystems, MDS Sciex, Concord, ON, Canada) at the Proteomics Unit of Complutense University of Madrid. The MALDI-TOF/TOF operated in positive reflector mode with an accelerating voltage of 20,000 V. All mass spectra were calibrated internally using peptides from the auto digestion of trypsin. The analysis by MALDI-TOF/TOF mass spectrometry produces peptide mass fingerprints (PMF) and the peptides observed with a signal-to-noise ratio greater than 10 can be collated and represented as a list of monoisotopic molecular weights and used to compare with the masses of theoretical trypsin digestion of sequences annotated in the protein data base. Proteins ambiguously identified by peptide mass fingerprints were subjected to MS/MS sequencing analyses. Thus, from the MS spectra, suitable precursors were selected for fragmentation analyses by CID (atmospheric gas was used) using a 1 Kv ion reflector method and precursor mass Windows +/− 4 Da. The plate model and default calibration were optimized for the MS-MS spectra processing. For protein identification, Allergome 20,200,317 (4402 sequences; 1,236,229 residues) without taxonomy restriction was searched using MASCOT 2.3 (www.matrixscience.com) through the software Global Protein Server v 3.6 (ABSciex, Concord, ON, Canada). The MASCOT search parameters were: (1) carbamidomethyl cysteine as fixed modification and oxidized methionine as variable modification; (2) peptide mass tolerance, 50 ppm (PMF) - 100 ppm (MSMS or combined search); (3) 1 missed trypsin cleavage site allowed; (4) MS-MS fragment tolerance, 0.3 Da.

### 3.6. Statistical Analysis

To compare the total and soluble protein content of treated and untreated (control) samples, both cashew and pistachio, a simple variance analysis (ANOVA) was carried out. Subsequently, a comparison of means was carried out using the Duncan test and significant differences were considered when *p* < 0.05. All analyzes were performed using the StatGraphic Centurion version XVII program (Graphics Software System, Rockville, MD, USA).

## 4. Conclusions

Thermal and pressure processing through controlled instant depressurization (DIC) affect the total content measured by RC-DC and considerably reduces the soluble protein content in both pistachio and cashew. Analysis of the electrophoretic profile of the control and treated flours of pistachio and cashew nut indicates that the number and intensity of bands is reduced in both as the pressure and DIC treatment time is increased. The greatest decrease is achieved with the most drastic treatment (DIC 7 bar, 120 s), although without reaching complete elimination in any of them. Immunodetection of potentially allergenic proteins with anti-2S and anti-11S IgG also decreases markedly after treatment at 7 bars and 120 s. Such treatment is more effective in pistachio than in cashew nut, with 11S legumes being more susceptible to degradation. Immunodetection with IgE of human sera reveals a clear individual variability at the level of the pattern of reactive bands. Almost all sera recognize 2S albumin of both pistachio and cashew nuts (Pis v 1 and Ana or 3) and some of them also recognize 11S legumins (Pis v 2 and Ana o 2). After DIC treatment at 7 bars and 120 s, the highest reduction in IgE binding is achieved for pistachio (75%) and cashew (67.2%). 

Therefore, pistachio and cashew have similar total and soluble protein but show differences in their electrophoretic and immunoreactive patterns against IgG and IgE antibodies. The DIC treatment at 7 bars during 120 s drastically reduces the allergenic capacity of both nuts, pistachio being more susceptible than cashew.

## Figures and Tables

**Table 1:** Total protein (LECO and RC-DC) and soluble (Bradford) in pistachio and cashew flours.

Sample	Total Protein (LECO) (g/100 g dm)	Total Protein (RC-DC) (g/100 g dm)	Soluble Protein (Bradford) (g/100 g dm)
Pistachio Raw	39.50 ± 0.14 ^a^ *	54.86 ± 0.00 ^a^	21.47 ± 1.24 ^a^
Pistachio DIC2	39.13 ± 0.01 ^a^	57.5 ± 3.17 ^a^	10.69 ± 1.14 ^b^
Pistachio DIC6	39.86 ± 0.28 ^ab^	37.99 ± 0.97 ^b^	5.84 ± 1.17 ^c^
Pistachio DIC8	38.48 ± 0.26 ^b^	30.91 ± 0.40 ^c^	3.86 ± 0.67 ^c^
Çashew Raw	35.43 ± 0.13 ^a^	55.04 ± 3.35 ^a^	23.24 ± 0.24 ^a^
Cashew DIC2	35.59 ± 0.38 ^a^	47.11 ± 1.06 ^b^	14.54 ± 0.33 ^b^
Cashew DIC6	33.74 ± 0.17 ^b^	33.2 ± 0.40 ^c^	2.80 ± 0.11 ^c^
Cashew DIC8	37.50 ± 0.18 ^c^	37.82 ± 1.50 ^c^	6.50 ± 0.14 ^d^

* Figures (means ± SE; *n* = 3) followed by different superscripts in the same column were significantly different (*p* < 0.05), compared by Duncan test.

**Table 2:** Immunological and clinical information from the 11 allergic patients included in this study.

Patient	Age/Sex	IgE Pistachio (kU/L)	IgE Cashew (kU/L)	Symptoms
P1	9/M	>100	>100	Anaphylaxis
P2	19/F	>100	>100	SWPC
P3	6/F	71	70.1	SWPC
P4	10/M	76.2	75.4	TS
P5	4/M	74.4	74.5	SWPC
P6	5/M	33.2	39.9	Urticaria, ES, C, D
P7	11/F	16.4	10.1	SWPC
P8	7/F	15.4	10.6	SWPC *
P9	5/F	29.3	23.4	Pruritus
P10	7/F	19.9	13.1	Anaphylaxis
P11	6/F	18.0	11.3	Urticaria, LS, ES

ES, eye swell; TS, tongue swell; D, dysphonia; C, cough; LS, lip swell; SWPC, sensitization without previous consumption; *, without access to clinical information.

**Table 3:** Conditions (pressure and time) of the DIC treatments initially selected from those applied in pistachio and cashew nuts. The treatments in bold are those selected for subsequent electrophoretic and immunodetection analysis.

Sample	Pressure (bar)	Time (s)
ST	--	--
DIC1	3.6	43
**DIC2**	**3.6**	**107**
DIC3	5	75
DIC4	5	120
DIC5	6.4	43
**DIC6**	**6.4**	**107**
DIC7	7	75
**DIC8**	**7**	**120**

## Supplementary Materials

The following are available online, Figure S1: IgE immunoblots with individual sera, Figure S2: IgE immunoreactive protein bands, Figure S3: SDS-PAGE (4-20%) of pistachio (A) and cashew (B), Table S1: Proteins separated by SDS-PAGE and identified by MALDI-TOF/TOF.
