# Supplementary material for: Influence of Instant Controlled Pressure Drop (DIC) on Allergenic Potential of Tree Nuts
Source: Molecules. 2020 Apr 10;25(7):1742. doi: 10.3390/molecules25071742 (PMC7180768; doi:10.3390/molecules25071742)

Figure S1. IgE immunoblots with individual sera. IgE immunoblot of proteins of pistachio A) and cashew (B) of untreated (control,ST) and DIC treated samples (20μg protein/lane). IgE immunoblots were carried out using individual sera from 11 patients allergic to pistachio and cashew (P1-P11). Lane C represents a negative control serum.

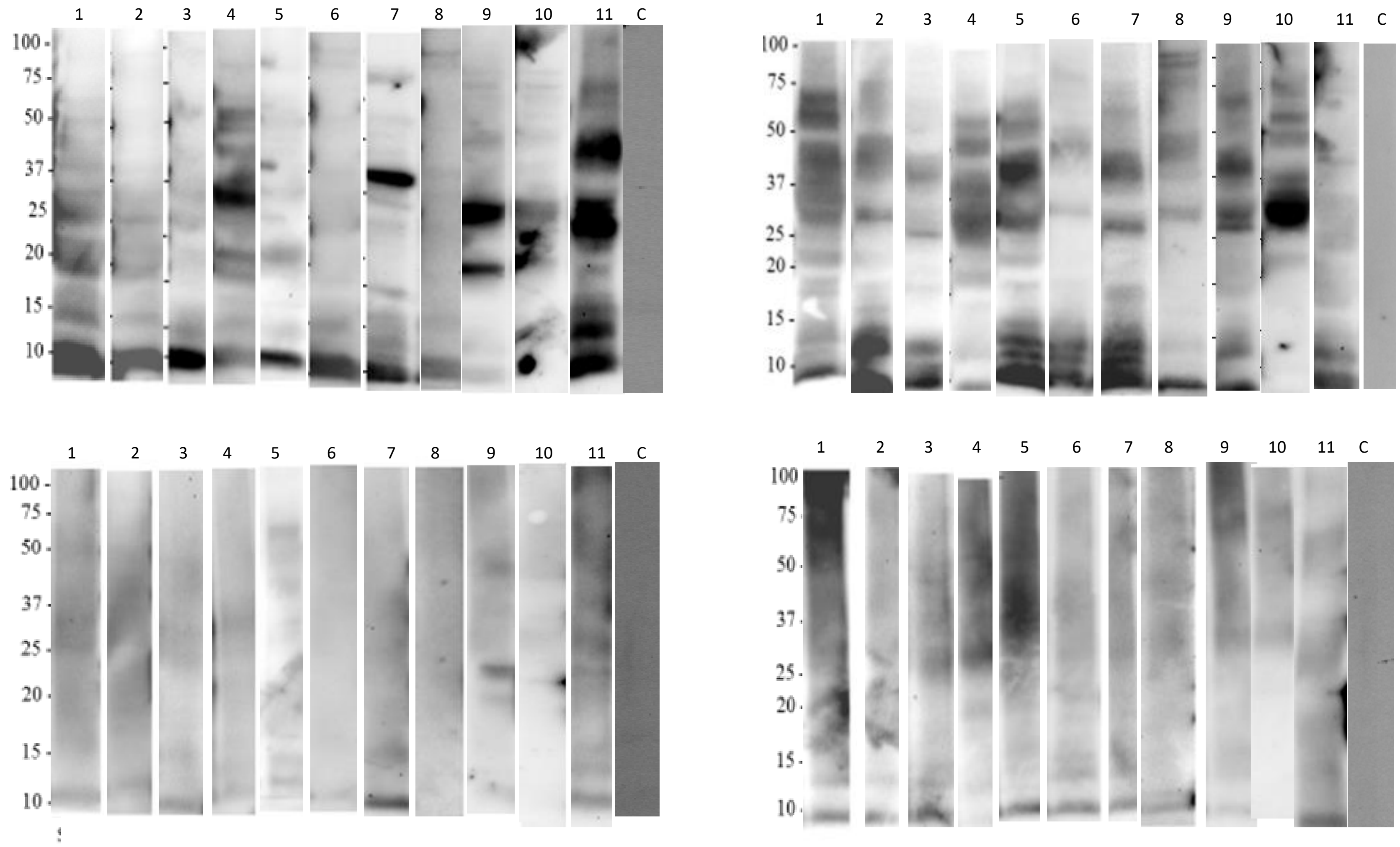

Figure S2. IgE immunoreactive protein bands. Percentage of patients that immunoreact with each protein band of pistachio (A) and cashew (B) untreated and treated by DIC 7 bar 120 seconds. Analysis performed with Quantity One Software (BioRad).

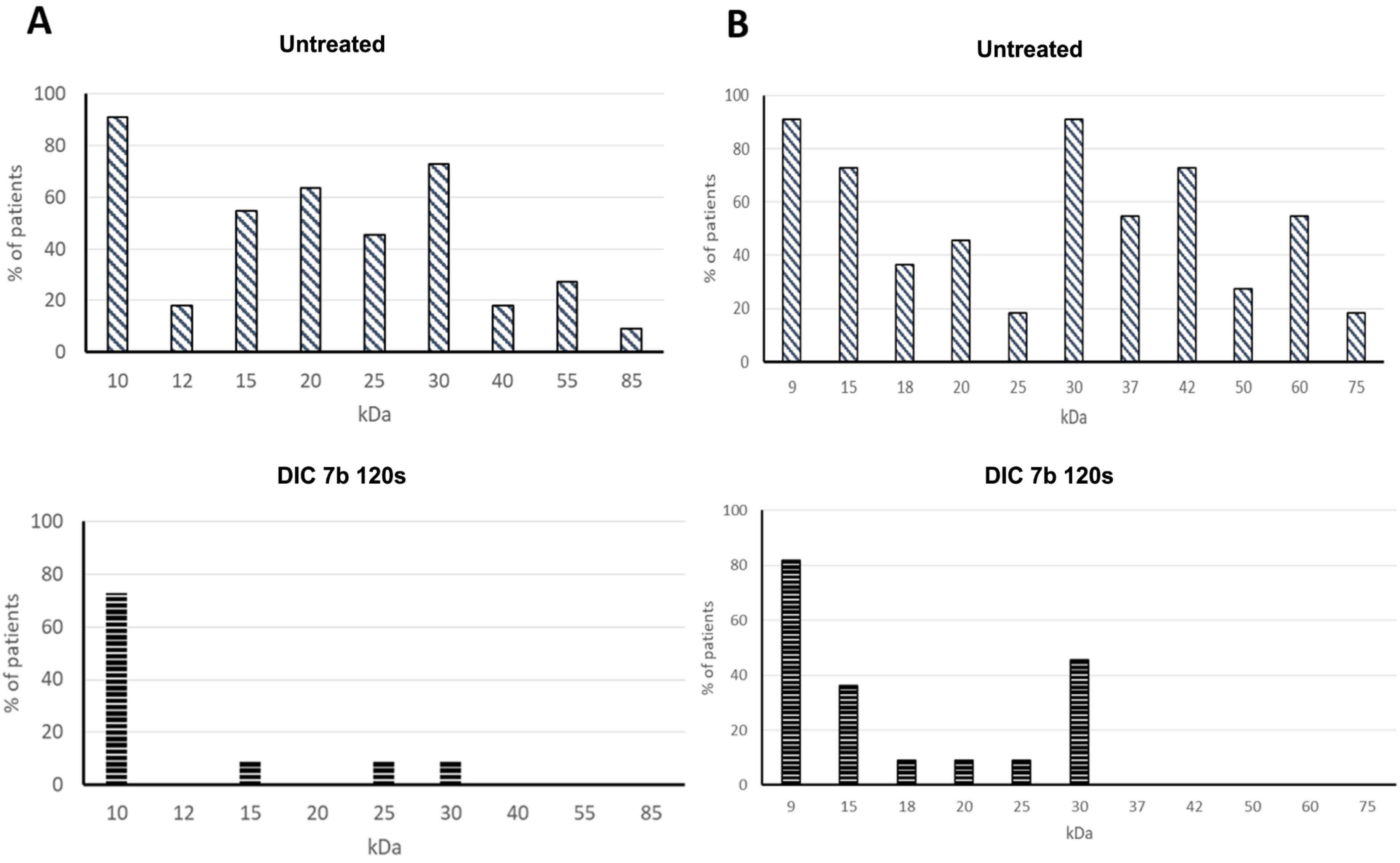

Supplement: Supplementary file 1 [file molecules-25-01742-s001.zip › Figure S1_S2 v3.pdf]
