# Supplementary material for: Influence of Instant Controlled Pressure Drop (DIC) on Allergenic Potential of Tree Nuts
Source: Molecules. 2020 Apr 10;25(7):1742. doi: 10.3390/molecules25071742 (PMC7180768; doi:10.3390/molecules25071742)
Supplement: Supplementary file 1 [file molecules-25-01742-s001.zip › Figure S3 maldi v3.pdf]

Figura S3. SDS-PAGE (4-20%) of pistachio (A) and cashew (B). Excised band proteins for identification are marked in red. (20µg proteín/lane)

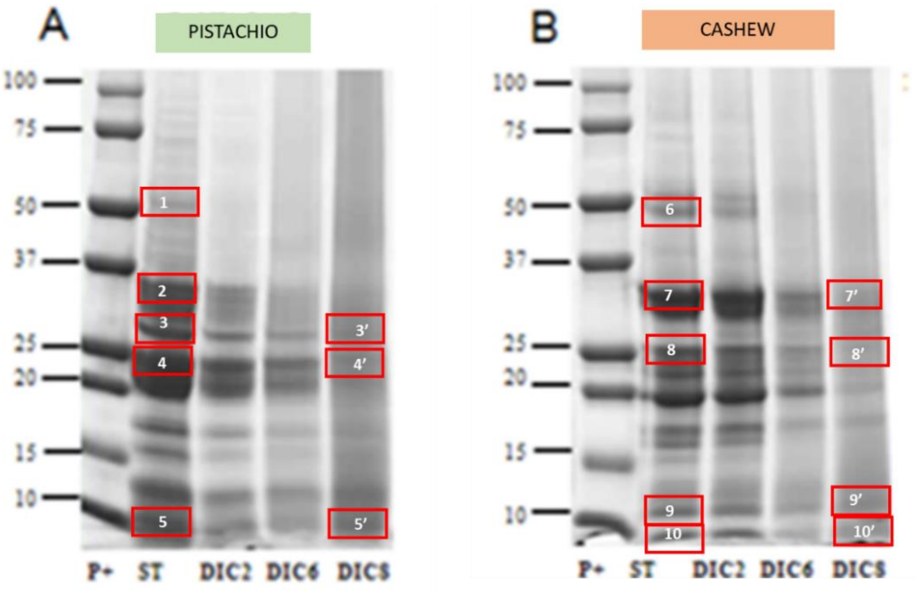

Table S1. Proteins separated by SDS-PAGE and identified by MALDI-TOF/TOF. Pistachio and cashew proteins from untreated and DIC8 treated samples (7 bar, 120 sec) identified by MALDI-TOF/TOF (PMF) or (PMF+MS-MS spectra)#

Band No.	No. access	Protein identification	Mascot score*	% Coverage	Mass (Da)	Matched peptides	Ion score
1	B7P073	Pis v 2.01 (11S *P.vera*)	215	51	56716	22	
1	B7SLJ1	Pis v 5.01/ (11S *P.vera*)	89	30	53896	13	
2	B4X640	Pis v 3.01 (7S *P.vera)*	94	26	60306	15	
2	B7SLJ1	Pis v 5.01 (11S *P.vera*)	54	18	53896	9	
3	B7P073	Pis v 2.01 (11S P.vera)	122	37	56716	14	
4	B7P073	Pis v 2.01 (11S *P.vera*)	159	45	56716	17	
5	B7P072	Pis v 1 (2S *P.vera)*	81	30	17792	9	
3'	B7P074	Pis v 2.02 /(11S *P.vera*)	103	38	53550	14	
4'	B7P073	Pis v 2.01 (11S *P.vera*)	124	34	56716	15	
**5'#**	B7P072	Pis v 1 (2S *P.vera*)	116	22	17792	--	74
6	Q8GZL6	Ana o 2.01 (11S *A. occidentale*)	79	25	52420	11	
6	Q8L5L6	Ana o 1.01 (7S *A. occidentale*)	68	26	62284	13	
7	Q8GZL6	Ana o 2.01 (11S *A. occidentale*)	79	24	52420	11	
8	Q8GZL6	Ana o 2.01 (11S *A. occidentale*)	49	35	52420	16	
9	Q8H2B8	Ana o 3 (2S *A. occidentale*)	59	55	16781	9	
**10#**	Q8H2B8	Ana o 3 (2S *A. occidentale*)	122	36	16781	--	61
7'	Q8GZL6	Ana o 2.01 (11S *A. occidentale*)	140	37	52420	18	
8'	Q8GZL6	Ana o 2.01 (11S *A. occidentale*)	150	34	52420	19	
9'	Q8GZL6	Ana o 2.01 (11S *A. occidentale*)	62	19	52,420	10	
10'	Q8H2B8	Ana o 3 (2S *A. occidentale*)	55	36	16,335	7	

* Protein scores greater than 49 are significant (P<0.05). Protein score is -10*Log (P), where P is the probability that the observed match is a random event.
